# Anti-Gametocyte Antigen Humoral Immunity and Gametocytemia During Treatment of Uncomplicated Falciparum Malaria: A Multi-National Study

**DOI:** 10.3389/fcimb.2022.804470

**Published:** 2022-04-07

**Authors:** Katherine O’Flaherty, Jo-Anne Chan, Julia C. Cutts, Sophie G. Zaloumis, Elizabeth A. Ashley, Aung Pyae Phyo, Damien R. Drew, Arjen M. Dondorp, Nicholas P. Day, Mehul Dhorda, Rick M. Fairhurst, Pharath Lim, Chanaki Amaratunga, Sasithon Pukrittayakamee, Tran Tinh Hien, Ye Htut, Mayfong Mayxay, M. Abul Faiz, Olugbenga A. Mokuolu, Marie A. Onyamboko, Caterina Fanello, Eizo Takashima, Takafumi Tsuboi, Michael Theisen, Francois Nosten, James G. Beeson, Julie A. Simpson, Nicholas J. White, Freya J. I. Fowkes

**Affiliations:** ^1^ Life Sciences, Burnet Institute, Melbourne, VIC, Australia; ^2^ Department of Medicine, The University of Melbourne, Melbourne, VIC, Australia; ^3^ Department of Immunology, Monash University, Melbourne, VIC, Australia; ^4^ Centre for Epidemiology and Biostatistics, School of Population and Global Health, The University of Melbourne, Melbourne, VIC, Australia; ^5^ Mahidol-Oxford Tropical Medicine Research Unit, Faculty of Tropical Medicine, Mahidol University, Bangkok, Thailand; ^6^ Centre for Tropical Medicine and Global Health, Nuffield Department of Medicine, University of Oxford, Oxford, United Kingdom; ^7^ Myanmar Oxford Clinical Research Unit, Yangon, Myanmar; ^8^ WorldWide Antimalarial Resistance Network, Asia-Pacific Regional Centre, Bangkok, Thailand; ^9^ Laboratory of Malaria and Vector Research, National Institute of Allergy and Infectious Diseases, National Institutes of Health, Rockville, MD, United States; ^10^ Faculty of Tropical Medicine, Mahidol University, Bangkok, Thailand; ^11^ Oxford University Clinical Research Unit, Hospital for Tropical Diseases, Ho Chi Minh City, Vietnam; ^12^ Department of Medical Research, Ministry of Health and Sports, Yangon, Myanmar; ^13^ Lao-Oxford-Mahosot Hospital-Wellcome Trust-Research Unit, Mahosot Hospital, Vientiane, Laos; ^14^ Institute of Research and Education Development, University of Health Sciences, Vientiane, Laos; ^15^ Malaria Research Group and Dev Care Foundation, Chittagong, Bangladesh; ^16^ Department of Paediatrics and Child Health, University of Ilorin, Ilorin, Nigeria; ^17^ Kinshasa School of Public Health, University of Kinshasa, Kinshasa, Democratic Republic of Congo; ^18^ Division of Malaria Research, Proteo-Science Center, Ehime University, Matsuyama, Japan; ^19^ Department for Congenital Disorders, Statens Serum Institut, Copenhagen, Denmark; ^20^ Centre for Medical Parasitology at Department of Immunology and Microbiology, University of Copenhagen, Copenhagen, Denmark; ^21^ Shoklo Malaria Research Unit, Mahidol-Oxford Tropical Medicine Research Unit, Faculty of Tropical Medicine, Mahidol University, Mae Sot, Thailand; ^22^ Department of Microbiology and Central Clinical School, Monash University, Melbourne, VIC, Australia; ^23^ Department of Infectious Diseases and Department of Epidemiology and Preventative Medicine, Monash University, Melbourne, VIC, Australia

**Keywords:** malaria, gametocyte, antibodies, falciparum malaria, clinical malaria, epidemiogy, immunity

## Abstract

**Introduction:**

Understanding the human immune response to *Plasmodium falciparum* gametocytes and its association with gametocytemia is essential for understanding the transmission of malaria as well as progressing transmission blocking vaccine candidates.

**Methods:**

In a multi-national clinical efficacy trial of artemisinin therapies (13 sites of varying transmission over South-East Asia and Africa), we measured Immunoglobulin G (IgG) responses to recombinant *P. falciparum* gametocyte antigens expressed on the gametocyte plasma membrane and leading transmission blocking vaccine candidates *Pf*s230 (*Pf*s230c and *Pf*s230D1M) and *Pf*s48/45 at enrolment in 1,114 participants with clinical falciparum malaria. Mixed effects linear and logistic regression were used to determine the association between gametocyte measures (gametocytemia and gametocyte density) and antibody outcomes at enrolment.

**Results:**

Microscopy detectable gametocytemia was observed in 11% (127/1,114) of participants at enrolment, and an additional 9% (95/1,114) over the follow-up period (up to day 42) (total 20% of participants [222/1,114]). IgG levels in response to *Pf*s230c, *Pf*s48/45 and *Pf*s230D1M varied across study sites at enrolment (*p < 0.001*), as did IgG seroprevalence for anti-*Pf*s230c and D1M IgG (*p < 0.001*), but not for anti-*Pf*s48/45 IgG (*p = 0.159*). In adjusted analyses, microscopy detectable gametocytemia at enrolment was associated with an increase in the odds of IgG seropositivity to the three gametocyte antigens (*Pf*s230c OR [95% CI], *p*: 1.70 [1.10, 2.62], *0.017*; *Pf*s48/45: 1.45 [0.85, 2.46], *0.174*; *Pf*s230D1M: 1.70 [1.03, 2.80], *0.037*), as was higher gametocyte density at enrolment (per two-fold change in gametocyte density *Pf*s230c OR [95% CI], *p*: 1.09 [1.02, 1.17], *0.008*; *Pf*s48/45: 1.05 [0.98, 1.13], *0.185*; *Pf*s230D1M: 1.07 [0.99, 1.14], *0.071*).

**Conclusion:**

*Pf*s230 and *Pf*s48/45 antibodies are naturally immunogenic targets associated with patent gametocytemia and increasing gametocyte density across multiple malaria endemic settings, including regions with emerging artemisinin-resistant *P. falciparum*.

## Introduction

Malaria control currently relies on prompt diagnosis and effective first-line antimalarial treatment with Artemisinin Combination Therapies (ACTs) and the use of insecticide treated bed nets. However, resistance to the artemisinin derivatives in *Plasmodium falciparum* is now firmly established within the Greater Mekong Sub-Region (GMS) of South-East Asia (Ashley et al., 2014; Imwong et al., 2020) and emerging in other regions (Uwimana et al., 2021), threating the management and control of malaria. To prevent the spread of artemisinin resistant *P. falciparum*, GMS countries have committed to the elimination of all species of human malaria by the year 2030 (WHO, 2016). The future development of vaccines that have reduce transmission, known as transmission-blocking vaccines, are regarded as a priority to achieve malaria elimination goals globally (Beeson et al., 2019).

Elimination of *P. falciparum*, including artemisinin resistant parasites, is dependent on preventing the transmission of sexual stage parasites (gametocytes) between human and mosquito. Patent gametocytemia (microscopy detectable) has been observed more frequently in clinical malaria patients with slow-clearing *P. falciparum* infections characteristic of artemisinin resistance (Ashley et al., 2014), which may enhance the spread of these strains in regions where artemisinin resistance has emerged. The success of malaria transmission may depend on several factors including the acquisition of human immunity to key gametocyte antigens. Naturally acquired immunity, which develops after repeated exposure to *Plasmodium* spp., has been shown to protect against clinical disease and high densities of blood-stage parasites. Antibodies specific for *P. falciparum* gametocytes also develop with age and repeated exposure, and their ability to prevent transmission is realised in the mosquito where they have been demonstrated to reduce fertilisation and further development of transmissible forms of the parasite (Bousema et al., 2006; Drakeley et al., 2006; Stone et al., 2016; Stone et al., 2018). Thus, the presence of anti-gametocyte antibodies may infer gametocytemia as well as of the onward transmission potential of *P. falciparum* parasites, including drug resistant parasites.

Gametocytes of *P. falciparum* undergo a complex process of development marked by several morphological stages that occur within infected erythrocytes in the human host. The early gametocyte-infected erythrocyte stages sequester in the bone marrow and the spleen, and evidence for development of antibodies to these stages is conflicted (Chan et al., 2018; Dantzler et al., 2019). Few responses to mature gametocyte-infected erythrocytes have been studied in detail, however, studies have demonstrated the acquisition of human immunity to key antigens present on the surface of mature gametocytes (van Dijk et al., 2001; Eksi et al., 2006). Antibodies do occur to antigens on the surface of the gametocyte-infected erythrocyte, but they appear to be much less prominent and occur at low levels (Chan et al., 2018; Dantzler et al., 2019). *Pf*s230 and *Pf*s48/45 are the most well characterised antibody targets of *P. falciparum* gametocytes, with *Pf*s230 currently in Phase II trials (Healy et al., 2021). They are essential for fertilisation in the mosquito midgut, and meta-analyses of six studies evaluating the transmission blocking role of anti-*Pf*s230 and *Pf*s48/45 antibodies (Drakeley et al., 2004; Bousema et al., 2006; Drakeley et al., 2006; van der Kolk et al., 2006; Bousema et al., 2007; Bousema et al., 2010) demonstrated that in ~57 - 69% of individuals these antibodies were shown to significantly reduce mosquito infection rates by >90% in standard membrane feeding assays (Stone et al., 2016). However, it is currently unknown how antibodies specific for gametocyte antigens vary within- and between different malaria endemic settings with varying gametocyte metrics. Here, we investigated the relationship between gametocytemia (prevalence and density at enrolment) and acquired anti- *Pf*s230 and *Pf*s48/45 antibody responses measured at enrolment in patients from South-East Asia and Africa participating a multinational trial of artemisinin treatment efficacy with varying rates of gametocytemia and artemisinin drug resistance.

## Methods

### Study Design and Procedures

Plasma samples were acquired from 1,114 participants from 11 South-East Asian study sites in Thailand (three sites: Mae Sot, Srisaket, Ranong), Cambodia (four sites: Pailin, Preah Vihear, Ratanakiri, Pursat), Bangladesh (Ramu), Myanmar (Shwe Kyin), Lao PDR (Attapeu) and Vietnam (Binh Phuoc), and two African sites; Democratic Republic of Congo (Kinshasa) and Nigeria (Ilorin), participating in a multicentre open label randomised trial of artemisinin mono- and combination treatment efficacy, described previously (Ashley et al., 2014). Participants were eligible for inclusion if they presented with uncomplicated falciparum malaria and were randomised to receive a 3-day course of either 2mg/kg or 4mg/kg artesunate monotherapy, followed by a full 3-day course of ACT. Peripheral asexual and gametocyte density was measured by microscopy at enrolment and 4, 6, 8, and 12 hours, then every 6 hours following treatment until two consecutive parasite negative blood slides were observed. Parasite counts were used to determine the primary outcome of the study, parasite clearance half-life (hours), derived using the WWARN parasite clearance estimator (Flegg et al., 2011). Follow up assessments were performed on days seven, 14, 28 and 42. Genotyping of recurrent or newly acquired infections and the *kelch*13 marker were performed as previously described (Ashley et al., 2014). Informed consent was obtained from all participants/legal guardians, and ethical approval was granted by national ethics committees in each participating country, the Oxford Tropical Research Ethics Committee (06/11) and Alfred Hospital Committee for Ethics, Australia (485/12).

### Recombinant Proteins

A truncated recombinant version of *Pf*s230, termed *Pf*s230D1M, based on the 3D7 allele was expressed in the mammalian HEK293 cell expression system as previously described (Chan et al., 2018). *Pf*s230D1M contains the first 6 cysteine domain of full-length protein (MacDonald et al., 2016), while *Pf*s230c contains the first 3 domains of full-length protein and expressed in a wheat germ cell-free expression system (Miura et al., 2013). Recombinant *Pf*s48/45 was produced in *L. lactis* and is a truncated form of full-length protein (Singh et al., 2015).

### Antibody Determination

Plasma samples acquired at enrolment were used to determine levels of IgG to recombinantly expressed gametocyte antigens by high-throughput ELISA (JANUS liquid handling system, Perkin Elmer) as previously described (Ataide et al., 2017). Briefly, Spectraplates were coated with recombinant *P. falciparum* gametocyte stage antigens *Pf*s230c, *Pf*s230D1M and *Pf*s48/45 (0.5μg/mL) and incubated overnight. Plates were blocked for 2 hours at room temperature with 1% casein in 1 x PBS, and then incubated with patient and control sera at dilutions optimised separately for each antigen construct (1:250 for *Pf*s230c and 1:800 for *Pf*s230D1M and *Pf*s48/45) in 0.1% casein PBS at room temperature for 2 hours. Final serum concentrations were selected based on sample reactivity and avoidance of high end optical density saturation following titration of a random subset of samples (n=39) for each antigen, separately (**Supplementary Figure 1**). Goat anti-human Horse Radish Peroxidase (HRP)-conjugated IgG antibody was added at a concentration of 1/2500 diluted in 0.1% casein in 1 x PBS for one hour at room temperature. Between each incubation and addition step, plates were washed three times with 1 x PBS + 0.05% Tween20 using an automated plate washer. ABTS substrate was added to each well and covered for 30 minutes at room temperature, then stopped with 1% SDS, and read in a spectrophotometer at 405nm. For each plate, a total of six wells were incubated with positive control sera consisting of pooled samples from highly reactive naturally exposed donors from Papua New Guinea. Additionally, on each plate a panel of negative control sera collected from unexposed Melbourne donors were incubated in six wells per plate. Wells containing no test sera were used to deduct background reactivity from each sample. A seropositivity cut-off point was set at an OD above the mean + 2SDs of a panel of Melbourne donors.

### Statistical Analyses

The distribution of the demographic, parasitological and antibody variables were described using median (25^th^-75^th^ percentiles, range) or frequency (95% Confidence Interval [CI]) where appropriate. Mann-Whitney tests were performed to compare medians, associations between categorical variables were assessed using chi-squared tests, and correlations between antibody responses were estimated by calculating Spearman’s Rho. Mixed effects linear and logistic regression was used to determine the effect of *kelch*13 on gametocyte outcomes (gametocytemia at enrolment and gametocytemia after treatment), including a random effect for study site. Mixed effects logistic regression was used to determine the effect of categorical (gametocyte smear positive/negative) and continuous (log_2_ gametocyte density [/μL]) gametocyte measures at enrolment on the odds of IgG seropositivity measured at enrolment, and mixed-effects linear regression was used for continuous antibody outcomes (IgG level [log_e_ optical density]) measured at enrolment. In addition, the effect of known risk factors for gametocytemia anaemia (hematocrit [%] at enrolment) and duration of symptoms [self-reported days of fever prior to enrolment]) on antibody outcomes was measured. All models assessing antibody measures as the outcome were adjusted for the potential confounder, age (years) and included a random effect for study site. Effect modification by the artemisinin resistance associated *kelch*13 genotype was assessed by comparing models with and without an interaction term between the *kelch*13 genotype and gametocytemia at enrolment using the likelihood ratio test. All analyses were performed using Stata version 15.

## Results

### Participant Characteristics and Gametocytemia

**Figure 1:**
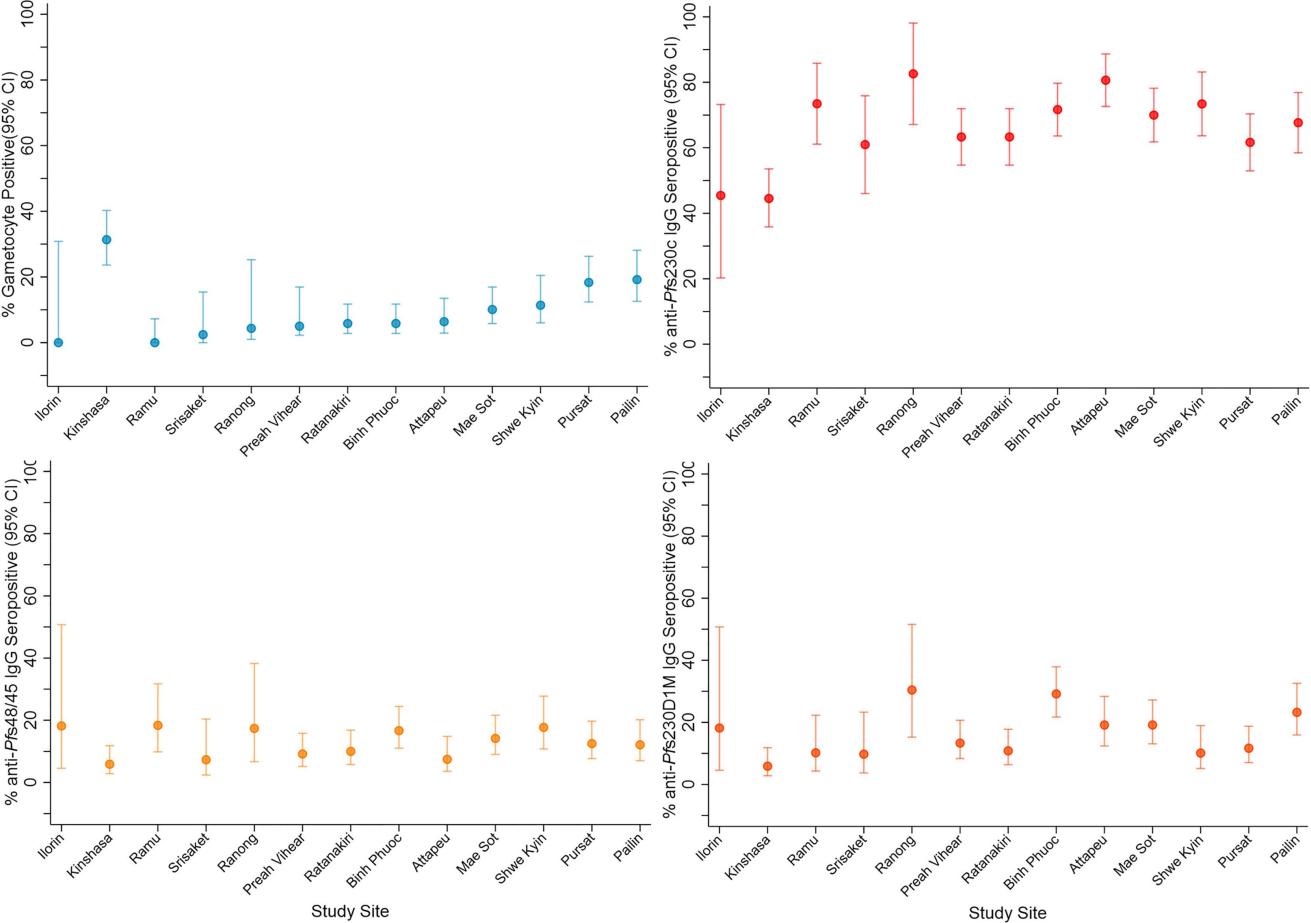
Gametocyte and IgG prevalence (95%CI) in response to gametocyte targets. IgG seroprevalence varied across study sites for *Pf*s230cand *Pf*s230D 1M (chi-squared test *p <*0.001) but not *Pf*s48/45 (chi-squared test *p* = 0.159). Study sites are arranged by continent (Africa - Nigeria (Ilorin n=11), Democratic Republic of Congo (Kinshasa n=119); Asia - Laos PDR (Attapeu n=93), Bangladesh (Ramu n=49), Thailand (Mae Sot n=120, Srisaket n=41, Ranong n=23), Cambodia (Pailin n=99, Preah Vihear n=120, Ratanakiri n=120, Pursat n=120), Myanmar (Shwe Kyin n=79), and Vietnam (Binh Phuoc n=120) and then in order of lowest to highest prevalence of gametocytemia at enrolment.

IgG antibodies were measured at enrolment in 1,114 patients participating in a clinical efficacy study of artemisinin derivatives across 13 study sites in South East Asia and Africa. Reflecting broad differences in malaria epidemiology between sites, participants recruited from South-East Asian sites were predominantly male adults (78.2%, [770/985]); median age 24 years [25^th^–75^th^ percentile: 18-34]), whereas participants recruited from African sites in Nigeria and the DRC, were all children (55% male (71/130); median age 4.5 years [25^th^–75^th^:3-6], **Table 1**). Microscopy detectable gametocytes were found in 11.4% (127/1,114) participants and median gametocyte density was 48 [16 – 192] gametocytes/μL at enrolment in gametocyte positive participants (**Table 2**). An additional 95 participants developed gametocytemia detected by microscopy in follow-up timepoints up until day 42, resulting in a total of 19.9% (222/1,114) of participants having any microscopy detectable gametocytemia throughout the study. The prevalence of gametocytemia at enrolment varied by study site, and was highest in Kinshasa, DRC (31.0% [37/118]) followed by Western Cambodian sites Pursat and Pailin (18.3% [22/120] and 19.2% [19/99], respectively) (**Table 2** and **Figure 1**), where artemisinin resistance is established and the proportion of participants infected with a *kelch*13 mutant strain was greatest (**Supplementary Table 1**) (Ashley et al., 2014). The odds of microscopy detectable gametocytemia was higher in participants infected with a *kelch*13 mutant *P. falciparum* strain compared to participants with a wild-type infection following treatment (Odds Ratio (OR) [95% Confidence Interval (CI)], *p value*: 2.39 [1.42, 4.03], *0.001*) but not at enrolment (OR [95% CI], *p value*: 0.86 [0.51, 1.45], *0.576*), nor was it associated with gametocyte density at enrolment or duration of microscopy detectable gametocytemia after treatment (**Supplementary Table 2**).

**Table 1:** Participant characteristics at each study site.

Country	Study Site	N	Age (years), Median (25^th^-75^th^ percentiles, min-max)	% Male (n/N)
**Africa**				
Nigeria	Ilorin	11	4 (2-6, 0.7-8)	73 (8/11)
DRC	Kinshasa	119	5 (3-6, 0.7-8)	53 (63/119)
**South-East Asia**				
Bangladesh	Ramu	49	26 (20–35, 10-55)	86 (42/49)
Cambodia	Pursat	120	25 (19–33, 3-60)	91 (109/120)
	Preah Vihear	120	20 (14–29, 4-58)	68 (82/120)
	Ratanakiri	120	14 (9-19.5, 2-55)	65 (78/120)
	Pailin	99	25 (19–38, 10-57)	87 (86/99)
Laos	Attapeu	93	23 (14–29, 6-60)	69 (64/93)
Myanmar	Shwe Kyin	79	24 (19-31, 1-54)	82 (65/79)
Thailand	Mae Sot	120	29 (22.5–37, 18-58)	78 (94/120)
	Srisaket	41	29 (22–38, 16-54)	100 (41/41)
	Ranong	23	33 (19-53)	70 (16/23)
Vietnam	Binh Phuoc	120	26 (18.5–38.5, 3-61)	77 (92/120)

**Table 2:** Gametocyte characteristics at each study site.

Country	Study Site	N	Gametocytemia at enrolment % (n/N)	Gametocyte density at enrolment^a^, Median (25^th^-75^th^ percentiles, min-max)	Gametocytemia during study period % (n/N)	Duration of gametocytemia^a^ (hours), Median (25^th^-75^th^ percentiles, min-max)
**Africa**						
Nigeria	Ilorin	11	0 (0/10)	–	0 (0/10)	–
DRC	Kinshasa	119	31 (37/118)	32 (16-80, 16-880)	46 (55/118)	72 (54-164, 2-335)
**South-East Asia**						
Bangladesh	Ramu	49	0 (0/49)	–	8 (4/49)	60 ^b^
Cambodia	Pursat	120	18 (22/120)	80 (16-496, 16-12058)	25 (30/120)	168 (84-330, 18-344)
	Preah Vihear	120	5 (6/120)	56 (32-176, 32-304)	8 (10/120)	156 (72-333, 42-357)
	Ratanakiri	120	6 (7/120)	64 (32-144, 16-9294)	8 (10/120)	168 (138-168, 48-311)
	Pailin	99	19 (19/99)	32 (16-176, 16-1200)	27 (27/99)	120 (66-308, 8-332)
Laos	Attapeu	93	6 (6/93)	136 (16-304, 16-336)	8 (7/93)	131 (73-144, 50-162)
Myanmar	Shwe Kyin	79	11 (9/79)	4019 (32-4898, 16-20010)	24 (19/79)	126 (56-168, 18-334)
Thailand	Mae Sot	120	10 (12/119)	64 (24-216, 16-3552)	27 (32/120)	116 (54-155, 6-335)
	Srisaket	41	2 (1/40)	384 (-, -)^b^	12 (5/41)	20 (12-56, 12-84)
	Ranong	23	4 (1/23)	48 (-, -)^b^	26 (6/23)	122 (108-161, 36-332)
Vietnam	Binh Phuoc	120	6 (7/120)	144 (48-576, 32-1296)	11 (13/120)	168 (96-337, 38-354)

a In gametocyte positive participants only.

b Only a single gametocyte positive participant.

### Between-Population Heterogeneity in Anti-Gametocyte Antigen IgG Responses

Total IgG levels and seroprevalence were determined at enrolment in response to the *P. falciparum* gametocyte antigens *Pf*s230c, *Pf*s230D1M and *Pf*s48/45. Overall, 65.8%, 15.7% and 11.9% of participants were categorised as seropositive for anti-*Pf*s230c anti-*Pf*s230D1M and anti-*Pf*s48/45 IgG, respectively. IgG seroprevalence varied between study sites for *Pf*s230c (range 44.5% - 82.6%, chi-squared test *p <0.001*) and *Pf*s230D1M (range 5.9% - 30.4%, chi-squared test *p <0.001*) (**Figure 1**). Comparatively, seroprevalence was lower for anti-*Pf*s48/45 IgG and did not vary significantly across sites (range 5.9% - 18.4%, chi-squared test *p = 0.159*) (**Figure 1**). Overall, there was a trend toward lower seroprevalence in Nigeria and DRC (range: 6% – 45%) compared to Asian study sites (range: 7% – 82%, **Figure 1**). Total IgG levels specific for all three antigens varied across study sites (Kruksall-Wallis *p <0.001*, **Supplementary Figure 2**). Total IgG levels in response to gametocyte antigens and previously published asexual stage antigens were moderately but significantly correlated (Rho range 0.1372 – 0.5395 all *p <0.05*, **Supplementary Table 3**) (Ataide et al., 2017).

### Quantifying the Association Between Gametocytemia and Anti-Gametocyte Antigen IgG

Seroprevalence of anti-gametocyte antigen IgG was greater in participants with microscopy detectable gametocytemia compared to gametocyte negative participants at enrolment in the majority of study sites (**Supplementary Figure 3**). In mixed-effects logistic regression (adjusted for age (years) and including a random effect for study site) microscopy detectable gametocytemia at enrolment was associated with a 45-70% increase in the odds of anti-gametocyte antigen IgG seropositivity (*Pf*s230c (OR) [95% (CI)], *p value*: 1.70 [1.10, 2.62], *0.017*; *Pf*s48/45: 1.45 [0.85, 2.46], *0.174*; *Pf*s230D1M: 1.70 [1.03, 2.80], *0.037*, **Table 3**). Additionally, a two-fold increase in gametocyte density at enrolment was associated with increases (5-9%) in the odds of IgG seropositivity (OR [95% CI], *p*, *Pf*s230c: 1.09 [1.02, 1.17], *0.008*; *Pf*s48/45: 1.05 [0.98, 1.13], *0.185*; *Pf*s230D1M: 1.07 [0.99, 1.14], *0.071*, **Table 3**). Similar associations between gametocytemia and levels of anti-gametocyte antigen IgG were also found (**Supplementary Table 4**). In addition, we also examined the association between hematocrit and duration of fever prior to enrolment and antibody outcomes, as these clinical variables are known to be associated with patent gametocytemia during uncomplicated *P. falciparum* malaria. Increasing hematocrit (%) was associated with a decrease in the odds of IgG seropositivity and a reduction in IgG level, and longer self-reported duration of fever prior to enrolment (days) was associated with an increase in the odds of IgG seropositivity and increased IgG levels (**Supplementary Table 5**), similar to their known associations with risk of patent gametocytemia (**Supplementary Table 6**).

**Table 3:** Effect of gametocytemia and gametocyte density on IgG seroprevalence at enrolment.

	OR (95% CI), *p*		
	*Pf*s230c	*Pf*s48/45	*Pf*s230D1M
**Gametocytemia at enrolment^a^**	1.70 (1.10, 2.62), *0.017*	1.45 (0.85, 2.46), *0.174*	1.70 (1.03, 2.80), *0.037*
**Gametocyte density at enrolment^b^**	1.09 (1.02, 1.17), *0.008*	1.05 (0.98, 1.13), *0.185*	1.07 (0.99, 1.14), *0.071*

OR – odds ratio, CI – confidence interval.

Estimates derived from mixed effects logistic regression adjusted for age (years) and specify a random effect for study site. ^a^Estimate for participants gametocyte positive compared to gametocyte negative patients at enrolment ^b^Estimate for a two-fold increase in gametocyte density (/μL).

To gain a greater understanding of the associations between gametocytemia and antibodies in the context of artemisinin resistant *P. falciparum* infections, we investigated whether these associations differed for participants infected with a wild-type or *kelch13* mutant *P. falciparum* strain. While the magnitude of association between microscopy detectable gametocytemia at enrolment and an increased odds of IgG seroprevalence was larger in participants infected with a *kelch13* mutant strain of *P. falciparum* (OR range 2.16-2.94) compared to those infected with a *kelch13* wild type strain (OR range 1.17-1.36), there was no evidence for effect modification by *kelch*13 genotype [*p* for interaction terms range *0.12* - *0.27* (**Table 4**)].

**Table 4:** Gametocytemia and odds of IgG seroprevalence according to kelch13 genotype.

	OR (95% CI), *p* ^a^		
	*Pf*s230c	*Pf*s48/45	*Pf*s230D1M
**Wild Type (n=743)**	1.36 (0.81, 2.27), *0.24*	1.17 (0.57, 2.38), *0.67*	1.35 (0.70, 2.62), *0.37*
**Mutant** **^b^** **(n=335)**	2.94 (1.24, 6.98), *0.02*	2.16 (0.95, 4.93), *0.07*	2.68 (1.24, 5.79), *0.01*

p-value for likelihood ratio tests: Pfs230c = 0.12, Pfs48/45 = 0.27, Pfs230D1M = 0.18.

OR, odds ratio; CI, confidence interval.

Estimates derived from mixed effects logistic regression including an interaction term between gametocytemia and kelch13 genotype, adjusted for age (years) and a random effect specified for study site.

a Odds of IgG seropositivity in participants gametocyte positive compared to gametocyte negative participants.

b Single point mutations in the propeller domain of kelch13 after position 440.

## Discussion

Antibodies specific for gametocyte stages may play a significant role in reducing the transmission of malaria. However, knowledge of the acquisition and prevalence of antibodies and how they relate to gametocytemia, and potential transmission of drug resistant *P. falciparum* is limited. We determined IgG levels and seroprevalence to the sexual stage *P. falciparum* antigens *Pf*s230 and *Pf*s48/45 in a multinational clinical efficacy trial of artemisinin therapy across study sites in South-East Asia and Africa, including regions of confirmed artemisinin resistance. Overall, anti-gametocyte antigen IgG responses varied significantly within and between study sites and were associated with microscopy detectable gametocytemia at enrolment and increased gametocyte density. Anti-gametocyte antigen antibodies may serve as markers of gametocyte exposure in malaria endemic populations, rather than biomarkers of active gametocytemia in individuals, and between study site variation suggests that the transmission potential of *P. falciparum* parasites, including drug resistant parasites, may vary between populations.

Identifying immunogenic gametocyte antigens is important for development of transmission blocking vaccines and could inform future development of serosurveillance tools. Seroprevalence of anti-*Pf*s230 IgG, but not *Pf*s48/45, varied by study site, which suggests that this antigen maybe more sensitive for delineating differences in gametocytemia and potential transmission blocking immunity across sites with varying transmission intensity. The observed reactivity and difference in seroprevalence across study sites may reflect differences in transmission intensity and prior exposure. Indeed, we have previously demonstrated significant variation in IgG responses specific for pre-erythrocytic and blood-stage parasite antigens in the same populations (Ataide et al., 2017), and anti-gametocyte antigen IgG was significantly correlated with these markers of transmission intensity and prior exposure in this study.

Anti-gametocyte antigen IgG seroprevalence was generally lower in participants recruited from DRC, a relatively higher transmission setting where patients were all children, but had the highest prevalence of gametocytes at enrolment, compared to participants recruited in Asian study sites, with relatively lower malaria transmission and where the majority of participants were adults. There may be several factors contributing to the lower seroprevalences observed in DRC which warrant further investigation. Firstly, despite the highest prevalence of gametocytemia at enrolment being observed in DRC, enrolment gametocyte density was generally lower compared to Asian study sites. Secondly, anti-gametocyte IgG responses have been shown to increase with age and exposure, as does their transmission reducing activity (Drakeley et al., 2006). Thirdly, geographical clustering of polymorphisms in gametocyte antigens has been demonstrated (Jones et al., 2015), which may differentially impact recognition of the gametocyte antigen constructs utilised across populations. Additionally, duration of gametocytemia, which may have differed between study sites, is likely to influence antibody acquisition and/or boosting, however, the duration of gametocytemia prior to clinical presentation and enrolment into the study is unknown. While the reasons for differential anti-gametocyte antigen IgG between DRC and Asian sites remain to be elucidated, our findings provide important data from Asian study sites and demonstrate that anti-gametocyte antigen responses are associated with patent circulating gametocytemia in low transmission settings. This is important given the paucity of studies assessing the prevalence of anti-gametocyte responses in low transmission settings when compared to studies in populations in moderate to high transmission settings (Stone et al., 2016).

Some patients had anti-gametocyte antigen antibodies in the absence of gametocytes, potentially a remnant from a previous infection or undetected gametocytes in the current infection. Microscopy misses low density gametocytemia and recent studies utilising molecular detection of sexual stages indicate that most infected individuals carry gametocytes (Schneider et al., 2007; Shekalaghe et al., 2007). The antibodies observed may be indicative of antibody boosting upon release of late-stage gametocytes at densities not detected by microscopy (i.e., at sub-microscopic densities). This hypothesis is supported by previous studies, which have observed rapid development of anti-gametocyte IgG responses following gametocyte exposure (Bousema et al., 2010; Skinner et al., 2015). Additionally, known risk factors of patent gametocytemia in clinical *P. falciparum* infection anaemia (estimated here using reduced hematocrit [%]), and increased duration of infection [duration of fever prior to enrolment (days)] (Price et al., 1999), which may act as proxy measures for gametocytemia during the current infection, were also associated with anti-gametocyte antigen antibodies. Finally, anti-asexual IgG responses were highly correlated with anti-gametocyte antigen responses in this study, which may further infer greater duration of infection in participants seropositive for and with greater levels of anti-gametocyte antigen IgG responses. Together, these findings further demonstrate that anti-gametocyte antigen IgG seropositivity is associated with patent gametocytemia and may act as a marker of exposure to gametocytemia within a population.

In this cohort, gametocytemia following treatment was higher in participants infected with a mutant *kelch*13 strain, and a systematic review has also demonstrated that artemisinin treatment failure is associated with a 15-fold greater risk of gametocytemia (WWARN, 2016). We observed greater odds of IgG seroprevalence with patent gametocytemia and increased gametocyte density at enrolment, however, we did not find statistically significant evidence of effect modification by *kelch*13 genotype on this association at enrolment despite biologically relevant differences in the odds of seroprevalence in genotype stratified analysis. Further investigation of this association in therapeutic efficacy trials following treatment are warranted to determine the impact of artemisinin resistance on gametocytemia and resulting antibody dynamics. While the transmission-blocking activity has been established for anti-*Pf*s230 and *Pf*s48/45 IgG antibodies (Stone et al., 2016; Stone et al., 2018), and residual infection following ACT treatment has been associated with longer gametocyte carriage and a subsequent greater risk of mosquito infection (Beshir et al., 2013), the ability of anti-gametocyte antigen antibodies to limit the onward transmission of artemisinin resistant *P. falciparum* across various transmission settings, however, is unknown and warrants further investigation.

A major strength of this study was that it included participants from diverse epidemiological settings across Africa and South-East Asia. The study enrolled patients with uncomplicated clinical malaria into a therapeutic efficacy study, and while they serve as sentinel surveillance populations for emerging drug resistance, the generalisability of our findings to individuals living in the community with asymptomatic malaria and gametocytemia is unknown. We included the two best characterised gametocyte antigens, which have demonstrated transmission blocking activity in animal models and by standard membrane feeding assay (Miura et al., 2013; Singh et al., 2015; MacDonald et al., 2016). However, the transmission blocking potential of antibodies measured in this clinical cohort were not directly quantified. Further, transmission blocking activity has been demonstrated in participant samples depleted of both anti-*Pf*s230 and *Pf*s48/45 specific IgG (Stone et al., 2018), implicating additional regions of the *Pf*s230 and *Pf*s48/45 proteins or further gametocyte targets in transmission blocking activity of human antibodies which warrant further investigation. Future studies of the relationship between gametocyte prevalence and dynamics of these and additional antibody responses longitudinally, as well transmission-blocking activity of these responses, particularly in asymptomatic populations, will further inform transmission potential of *P. falciparum*, including drug resistant parasites.

## Conclusions

In a multinational therapeutic efficacy trial of artemisinins in clinical malaria patients, we found antibodies against gametocyte antigens *Pf*s230c, *Pf*s230D1M and *Pf*s48/45 were associated with patent gametocytemia across populations from varying malaria endemicity. These findings further our understanding of acquired antibody responses to gametocytes, advancing deepening our understanding of antibody responses to progress transmission-blocking vaccine candidates.

## Data Availability Statement

The datasets presented in this article are not readily available because reasonable requests to the access of study data will be considered upon application to the corresponding authors. De-identified, individual participant data will be available to researchers whose proposed purpose of use is approved by the data access committee at Mahidol Oxford Tropical Medicine Research Unit. Inquiries or requests for the data may be sent to datasharing@tropmedres.ac. Requests to access the datasets should be directed to katherine.oflaherty@burnet.edu.au or freya.fowkes@burnet.edu.au.

## Ethics Statement

The studies involving human participants were reviewed and approved by Alfred Hospital Human Research and Ethics Committee, Australia, the Oxford Tropical Research Ethics Committee, United Kingdom, and relevant national ethics committees (Thailand: Ethics Committee of the Faculty of Tropical Medicine, Mahidol University and Tak Province Community Ethics Advisory Board (T-CAB), Cambodia: National Ethics Committee for Health Research, Ministry of Health, Kingdom of Cambodia Institutional Review Board and National Institute of Allergy and Infectious Diseases, Bethesda, Maryland, USA, Myanmar: The Government of the Republic of the Union of Myanmar, Ministry of Health, Department of Medical Research (Lower Myanmar), Laos: Ministry of Health. National Ethics Committee for Health Research, Lao Peoples’ Democratic Republic, Nigeria: Ethical Review Committee, University of Ilorin Teaching Hospital, Ilorin, Nigeria, Bangladesh: National Research Ethics Committee, Bangladesh Medical Research Council, Democratic Republic of the Congo: Republique Democratique du Congo, Ministere de l’Enseignement Superieur, Universitaire et Recherche Scientifique, Universite de Kinshasa, Ecole de Sante Publique Comite d’Ethique and Viet Nam: Ethics Committee for biomedical research of the Ministry of Health, Institute of Malariology-Parasitology-Entomology, Ho Chi Minh City). Written informed consent to participate in this study was provided by all participants/participants legal guardian/next of kin.

## Author Contributions

EA, FN, JS, NW, JB, and FF designed the research protocol. KO’F, J-AC, JC, SZ, AP, DD, AD, ND, MD, RF, PL, CA, SP, TH, YH, MM, MF, OM, MO, CF, FN, JB, JS, NW, and FF performed the research, J-AC, DD, ET, TT, MT, JB, and FF provided reagents/analytical tools. KO’F, JC, JS, and FF wrote the manuscript. All authors provided critical revision to the manuscript and have approved the final version.

## Funding

This work was supported by the National Health and Medical Research Council of Australia (project grant 1060785 [to FF, JS, and FN], program grant 637406 [to JB], 1166753 Career Development Fellowship [to FF], and investigator grants 1173046 [to JB] and 1196068 [to JS]), the Australian Centre for Research Excellence in Malaria Elimination (1134989), the Ramaciotti Foundation (Establishment Grant 3245/2011), the Ian Potter Foundation (grant to FF), the Victorian State Government (Operational Infrastructure Support grant), the United Kingdom Department for International Development, with additional support from the Worldwide Antimalarial Resistance Network, and the Intramural Research Program of the National Institute of Allergy and Infectious Diseases, National Institutes of Health, and the Wellcome Trust of Great Britain (support to the Wellcome Trust Mahidol Oxford Tropical Medicine Research Programme). This research was funded in part by the Wellcome Trust [220211]. For the purpose of Open Access, the author has applied a CC BY public copyright licence to any Author Accepted Manuscript version arising from this submission. The funders had no role in the study design, data collection and analysis, decision to publish or preparation of the manuscript.

## Conflict of Interest

J-AC served as a guest editor for Frontiers in Cellular and Infection Microbiology.

The remaining authors declare that the research was conducted in the absence of any commercial or financial relationships that could be construed as a potential conflict of interest.

## Publisher’s Note

All claims expressed in this article are solely those of the authors and do not necessarily represent those of their affiliated organizations, or those of the publisher, the editors and the reviewers. Any product that may be evaluated in this article, or claim that may be made by its manufacturer, is not guaranteed or endorsed by the publisher.

## Supplementary Material

Supplementary Figure 1 Serum dilution optimisation for anti-*Pf*s230D1M, *Pf*s48/45 and *Pf*s230C IgG in a random sub-sample of participant samples (n = 39).

Supplementary Figure 2 Gametocyte density at enrolment (log_e_ transformed median, 25^th^ and 75^th^ percentiles) and IgG level (log_2_ transformed median, 25^th^ and 75^th^ percentiles) in response to gametocyte targets *Pf*s230c, *Pf*s48/45 and *Pf*s230D 1M. IgG level varied by study site (Kruksall-Wallis *p<0.001*). Study sites are arranged by continent (Africa - Nigeria (Ilorin n=11), Democratic Republic of Congo (Kinshasa n=119); Asia - Laos PDR (Attapeu n=93), Bangladesh (Ramu n=49), Thailand (Mae Sot n=120, Srisaket n=41, Ranong n=23), Cambodia (Pailin n=99, Preah Vihear n=120, Ratanakiri n=120, Pursat n=120), Myanmar (Shwe Kyin n=79), and Vietnam (Binh Phuoc n=120) and then in order of lowest to highest prevalence of gametocytemia at enrolment.

Supplementary Figure 3 IgG seroprevalence (95% CI) in participants negative (red) and positive (orange) for gametocytes at enrolment. Study sites are arranged by continent (Africa - Nigeria (Ilorin n=11), Democratic Republic of Congo (Kinshasa n=119); Asia - Laos PDR (Attapeu n=93), Bangladesh (Ramu n=49), Thailand (Mae Sot n=120, Srisaket n=41, Ranong n=23), Cambodia (Pailin n=99, Preah Vihear n=120, Ratanakiri n=120, Pursat n=120), Myanmar (Shwe Kyin n=79), and Vietnam (Binh Phuoc n=120)) and then in order of lowest to highest prevalence of gametocytemia at enrolment.
