# Supplementary figures and images for: Anti-Gametocyte Antigen Humoral Immunity and Gametocytemia During Treatment of Uncomplicated Falciparum Malaria: A Multi-National Study
Source: Front Cell Infect Microbiol. 2022 Apr 7;12:804470. doi: 10.3389/fcimb.2022.804470 (PMC9022117; doi:10.3389/fcimb.2022.804470)

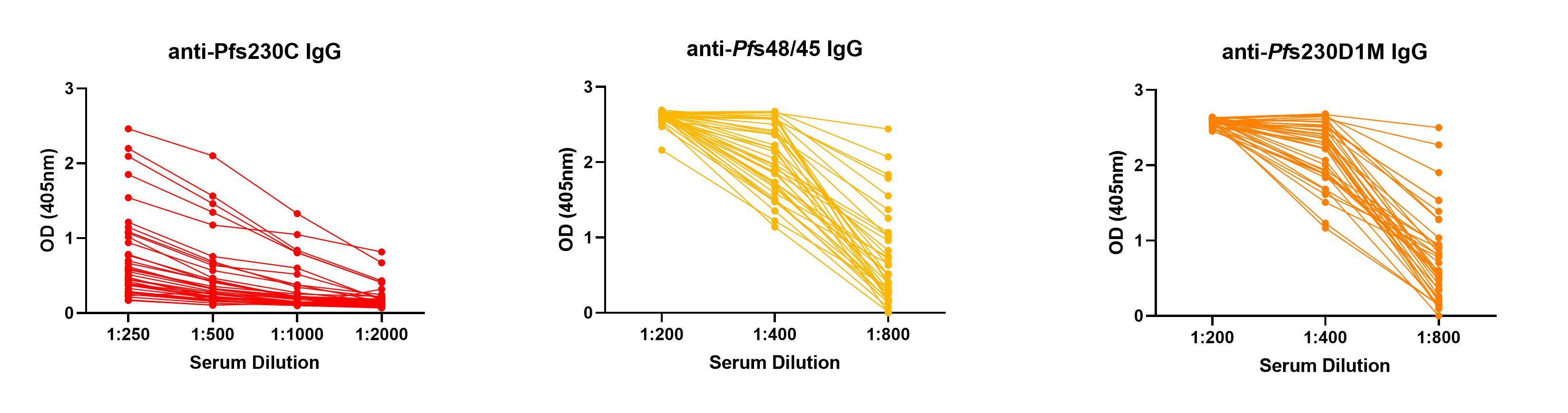

Supplement: Supplementary Figure 1 — Serum dilution optimisation for anti-Pfs230D1M, Pfs48/45 and Pfs230C IgG in a random sub-sample of participant samples (n = 39). [file Image_1.jpg]

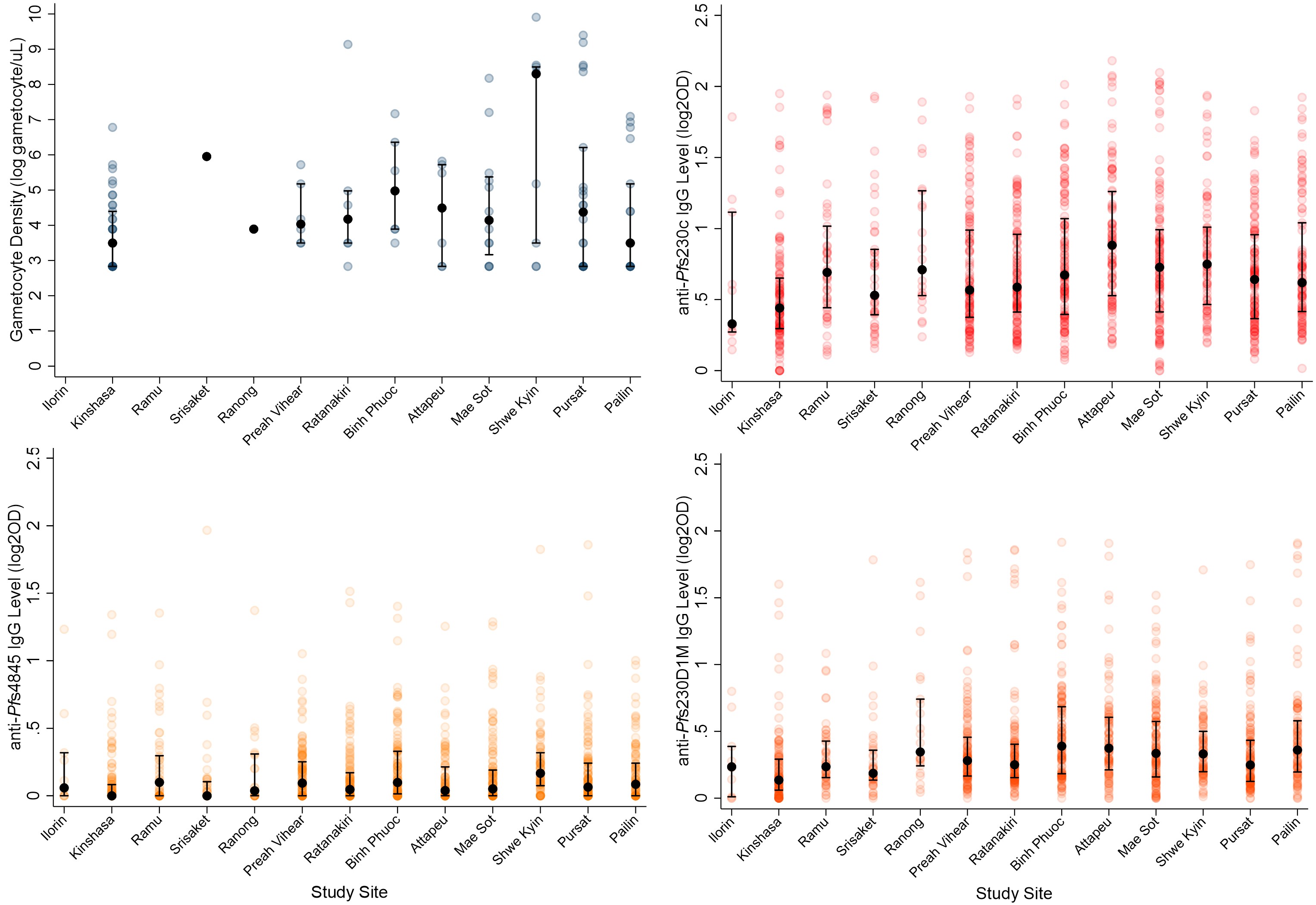

Supplement: Supplementary Figure 2 — Gametocyte density at enrolment (loge transformed median, 25th and 75th percentiles) and IgG level (log2 transformed median, 25th and 75th percentiles) in response to gametocyte targets Pfs230c, Pfs48/45 and Pfs230D 1M. IgG level varied by study site (Kruksall-Wallis p<0.001). Study sites are arranged by continent (Africa - Nigeria (Ilorin n=11), Democratic Republic of Congo (Kinshasa n=119); Asia - Laos PDR (Attapeu n=93), Bangladesh (Ramu n=49), Thailand (Mae Sot n=120, Srisaket n=41, Ranong n=23), Cambodia (Pailin n=99, Preah Vihear n=120, Ratanakiri n=120, Pursat n=120), Myanmar (Shwe Kyin n=79), and Vietnam (Binh Phuoc n=120) and then in order of lowest to highest prevalence of gametocytemia at enrolment. [file Image_2.jpeg]

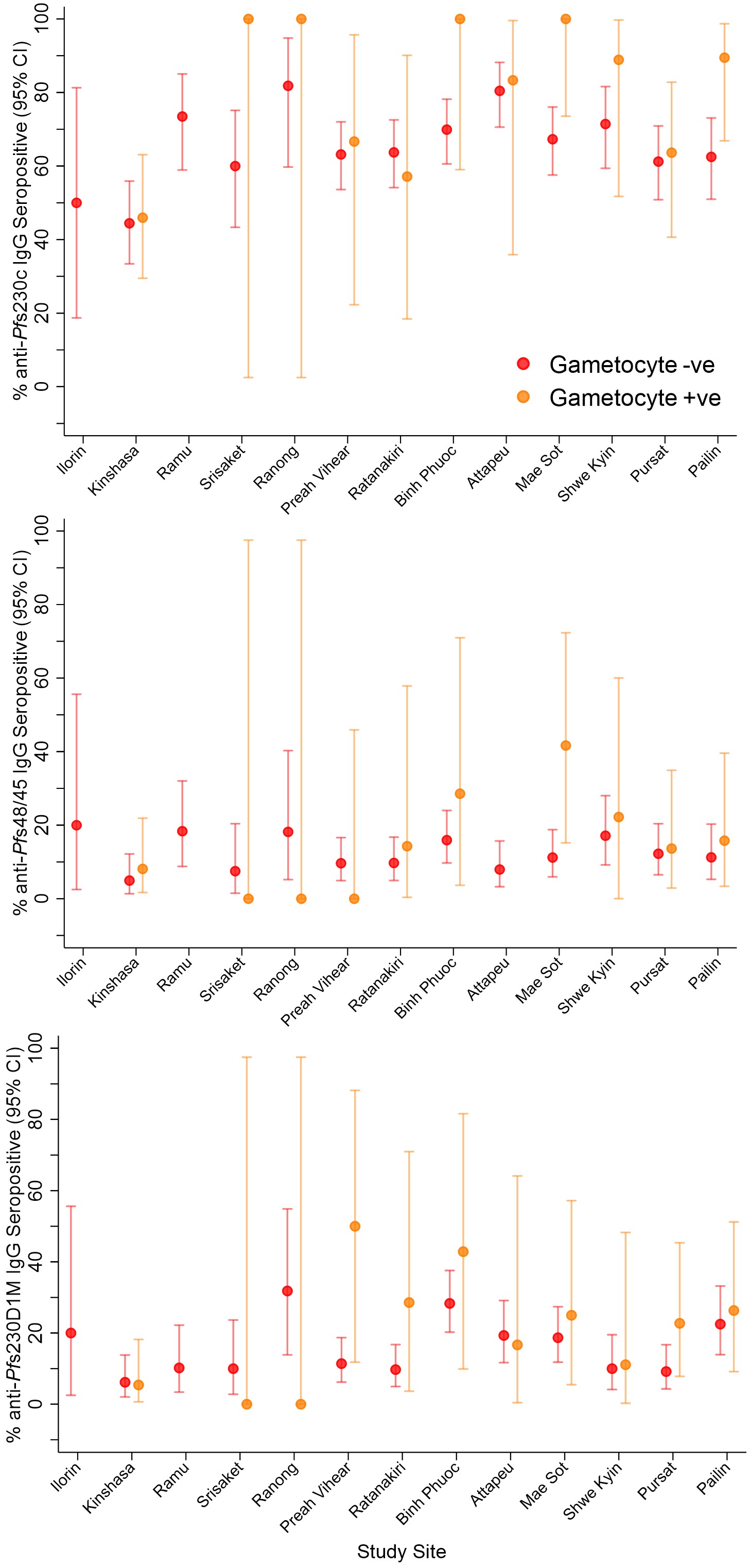

Supplement: Supplementary Figure 3 — IgG seroprevalence (95% CI) in participants negative (red) and positive (orange) for gametocytes at enrolment. Study sites are arranged by continent (Africa - Nigeria (Ilorin n=11), Democratic Republic of Congo (Kinshasa n=119); Asia - Laos PDR (Attapeu n=93), Bangladesh (Ramu n=49), Thailand (Mae Sot n=120, Srisaket n=41, Ranong n=23), Cambodia (Pailin n=99, Preah Vihear n=120, Ratanakiri n=120, Pursat n=120), Myanmar (Shwe Kyin n=79), and Vietnam (Binh Phuoc n=120)) and then in order of lowest to highest prevalence of gametocytemia at enrolment. [file Image_3.jpeg]
