# Supplementary material for: Anti-Gametocyte Antigen Humoral Immunity and Gametocytemia During Treatment of Uncomplicated Falciparum Malaria: A Multi-National Study
Source: Front Cell Infect Microbiol. 2022 Apr 7;12:804470. doi: 10.3389/fcimb.2022.804470 (PMC9022117; doi:10.3389/fcimb.2022.804470)
Supplement: Supplementary file 4 [file Table_1.docx]

| **Supplementary Table 1: Characteristics and treatment efficacy outcomes of study participants** | | | | | | | |  |
| --- | --- | --- | --- | --- | --- | --- | --- | --- |
| **Country** | **Study Site** | | **N** | **PCt_½_ (hours)**  **Median (25^th^-75^th^ percentiles, min-max)** | **% PCt_½_ ≥ 5 hours**  **(n/N)** | **% Parasitemic at Day 3**  **(n/N)** | **% *kelch13* mutants** | |
| **Africa** | | | | | | | | |
| Nigeria | Ilorin | | 11 | 2.1 (2.1-2.8, 1.4-7.1) | 20 (1/5) | 20 (1/5) | 0 (0/8) | |
| DRC | Kinshasa | | 119 | 2 (1.6-2.4, 0.7-7) | 2 (2/118) | 1 (1/118) | 3 (3/119) | |
| **South-East Asia** | | | | | | | | |
| Bangladesh | Ramu | | 49 | 2.6 (2-3.2, 0.7-5.4) | 2 (1/49) | 2 (1/48) | 0 (0/49) | |
| Cambodia | Pursat | | 120 | 5.6 (4.3-6.7, 1.7-11.8) | 61 (73/119) | 71 (85/119) | 66 (76/115) | |
|  | Preah Vihear | | 120 | 3 (2.5-4.2, 1.2-12.6) | 22 (26/120) | 24 (29/120) | 19 (22/113) | |
|  | Ratanikiri | | 120 | 3 (2.3-3.5, 0.7-8.8) | 6 (7/120) | 9 (11/118) | 3 (4/116) | |
|  | Pailin | | 99 | 6.1 (4.9-7.2, 2.4-9) | 74 (71/96) | 73 (71/97) | 80 (79/99) | |
| Laos | Attapeu | | 93 | 2 (1.6-2.7, 1.1-9.2) | 6 (5/84) | 11 (10/92) | 3 (3/92) | |
| Myanmar | Shwe Kyin | | 79 | 3.1 (2.6-4.1, 1.3-8.6) | 13 (10/77) | 15 (12/78) | 22 (17/77) | |
| Thailand | Mae Sot | | 120 | 4.9 (3.7-6.4, 0.6-10.1) | 50 (58/117) | 45 (53/118) | 51 (60/117) | |
|  | Srisaket | | 41 | 7.0 (4.3-8.7, 1.6-13.9) | 67 (24/36) | 68 (23/34) | 83 (30/36) | |
|  | Ranong | | 23 | 5.3 (3.5-6.4, 2.4-13.8) | 59 (13/22) | 60 (12/20) | 65 (13/20) | |
| Vietnam | Binh Phuoc | | 120 | 3.1 (1.9-5.3, 0.7-8.9) | 28 (33/118) | 32 (38/118) | 24 (28/117) | |
|  | |  | | | | | |  |
