# Supplementary material for: Anti-Gametocyte Antigen Humoral Immunity and Gametocytemia During Treatment of Uncomplicated Falciparum Malaria: A Multi-National Study
Source: Front Cell Infect Microbiol. 2022 Apr 7;12:804470. doi: 10.3389/fcimb.2022.804470 (PMC9022117; doi:10.3389/fcimb.2022.804470)
Supplement: Supplementary file 5 [file Table_2.docx]

| **Supplementary Table 2: Effect *kelch*13 genotype on gametocyte outcomes** | |
| --- | --- |
|  | **Odds of gametocytemia at enrolment**  **OR (95% CI), *p*** |
| ***kelch*13 mutant ^a^** | 0.86 (0.51, 1.45), *0.576* |
|  | **Mean difference in log_e_ gametocyte density at enrolment (95% CI), *p*** |
|  | -0.12 (-0.35, 0.11), *0.314* |
|  | **Odds of gametocytemia following treatment**  **OR (95% CI), *p*** |
|  | 2.39 (1.42, 4.03), *0.001* |
|  | **Mean difference in duration of detectable gametocytemia (hours)** **(95% CI), *p*** |
|  | -25.82 (-61.65, 10.92), *0.158* |
| OR – odds ratio, CI - confidence interval  Estimates derived from mixed effects logistic and linear regression adjusted for age (years) and a random effect specified for study site.  ^a^ Estimate for participants infected with a *kelch*13 mutant *P. falciparum* strain compared to participants infected with a *kelch*13 wild type *P. falciparum* strain | |
