# Supplementary material for: Anti-Gametocyte Antigen Humoral Immunity and Gametocytemia During Treatment of Uncomplicated Falciparum Malaria: A Multi-National Study
Source: Front Cell Infect Microbiol. 2022 Apr 7;12:804470. doi: 10.3389/fcimb.2022.804470 (PMC9022117; doi:10.3389/fcimb.2022.804470)
Supplement: Supplementary file 6 [file Table_3.docx]

| **Supplementary Table 3: Spearman’s Rho* correlation between sexual and asexual IgG responses (log_2_ optical density)** | | | |  |
| --- | --- | --- | --- | --- |
| **IgG Response** | ***Pf*s230C** | ***Pf*s48/45** | ***Pf*s230D1M** | |
| ***Pf*s230C** | 1 |  |  | |
| ***Pf*s48/45** | 0.3887 | 1 |  | |
| ***Pf*s230D1M** | 0.4954 | 0.5395 | 1 | |
| **MSP2 3D7** | 0.4327 | 0.3365 | 0.3561 | |
| ***Pf*EMP1 (VSA)** | 0.2806 | 0.1610 | 0.1372 | |
| **Rh2** | 0.4603 | 0.3819 | 0.3483 | |
| **MSP2 FC27** | 0.4857 | 0.3571 | 0.3840 | |
| **AMA1** | 0.4025 | 0.3120 | 0.2653 | |
| **CSP** | 0.5291 | 0.3366 | 0.4106 | |
| **MSP1** | 0.3224 | 0.3030 | 0.2992 | |
| **EBA-175 RII** | 0.3944 | 0.2889 | 0.2588 | |
| **MSP3** | 0.4678 | 0.3671 | 0.3090 | |
| **MSP7** | 0.4439 | 0.3356 | 0.2903 | |
| **EBA175 RIII-V** | 0.5387 | 0.3798 | 0.3562 | |
| *all *p < 0.001* | | | |  |
