# Supplementary material for: Anti-Gametocyte Antigen Humoral Immunity and Gametocytemia During Treatment of Uncomplicated Falciparum Malaria: A Multi-National Study
Source: Front Cell Infect Microbiol. 2022 Apr 7;12:804470. doi: 10.3389/fcimb.2022.804470 (PMC9022117; doi:10.3389/fcimb.2022.804470)
Supplement: Supplementary file 7 [file Table_4.docx]

| **Supplementary Table 4: Effect of gametocytemia and gametocyte density on IgG level at enrolment** | | | | | | |
| --- | --- | --- | --- | --- | --- | --- |
|  | **Relative change in geometric mean IgG level (log_e_ OD)**  **(95% CI), *p*** | | | | | |
|  | ***Pf*s230c** | | ***Pf*s48/45** | | ***Pf*s230D1M** | |
| **Gametocytemia at enrolment** ^a^ | 1.11 (1.05, 1.17), *<0.001* | 1.02 (0.99, 1.06), *0.149* | | 1.09 (1.04, 1.14), *<0.001* | |  |
| **Gametocyte density at enrolment ^b^** | 1.01 (1.00, 1.02), *0.003* | 1.00 (0.99, 1.01), *0.201* | | 1.01 (1.01, 1.02), *<0.001* | |  |
| OD – optical density, CI – confidence interval  Estimates derived from mixed effects linear regression adjusted for age (years) and specify a random effect for study site.  ^a^ Estimate for participants gametocyte positive compared to gametocyte negative patients at enrolment ^b^ Estimate for a two-fold increase in gametocyte density (/μL) | | | | | | |
