# Supplementary material for: Anti-Gametocyte Antigen Humoral Immunity and Gametocytemia During Treatment of Uncomplicated Falciparum Malaria: A Multi-National Study
Source: Front Cell Infect Microbiol. 2022 Apr 7;12:804470. doi: 10.3389/fcimb.2022.804470 (PMC9022117; doi:10.3389/fcimb.2022.804470)
Supplement: Supplementary file 8 [file Table_5.docx]

| **Supplementary Table 5: Effect of enrolment Hct (%) and duration of fever prior to enrolment (days) on IgG outcomes** | | | |
| --- | --- | --- | --- |
|  | **Odds of IgG seroprevalence**  **Odds Ratio (95% CI), *p*** | | |
|  | ***Pf*s230c** | ***Pf*s48/45** | ***Pf*s230D1M** |
| **Hct (%) at enrolment ^a^** | 0.95 (0.93, 0.98), *<0.001* | 0.95 (0.92, 0.98), *<0.001* | 0.96 (0.93, 0.99), *<0.001* |
| **Duration of fever prior to enrolment (days) ^b^** | 1.06 (0.98, 1.15), *0.160* | 1.22 (1.11, 1.34), *<0.001* | 1.21 (1.10, 1.33), *<0.001* |
|  | **Relative change in geometric mean IgG level (log_e_ OD)**  **(95% CI), *p*** | | |
|  | ***Pf*s230c** | ***Pf*s48/45** | ***Pf*s230D1M** |
| **Hct (%) at enrolment ^a^** | 0.99 (0.99, 1.00), <*0.001* | 1.00 (0.99, 1.00), <*0.001* | 1.00 (0.99, 1.00), *<0.001* |
| **Duration of fever prior to enrolment (days) ^b^** | 1.02 (1.01, 1.04), *<0.001* | 1.02 (1.01, 1.02), <*0.001* | 1.02 (1.01, 1.03), *<0.001* |
| Estimates derived from mixed effects linear and logistic regression, adjusted for age (years) and a random effect specified for study site.  ^a^ Estimate for a 1% increase in Hct ^b^ Estimate for a one day increase in pre-enrolment fever duration | | | |
