# Supplementary material for: Anti-Gametocyte Antigen Humoral Immunity and Gametocytemia During Treatment of Uncomplicated Falciparum Malaria: A Multi-National Study
Source: Front Cell Infect Microbiol. 2022 Apr 7;12:804470. doi: 10.3389/fcimb.2022.804470 (PMC9022117; doi:10.3389/fcimb.2022.804470)
Supplement: Supplementary file 9 [file Table_6.docx]

| **Supplementary Table 6: Effect of enrolment Hct (%) and duration of fever prior to enrolment (days) on gametocyte outcomes** | |
| --- | --- |
|  | **Odds of gametocytemia at enrolment**  **Odds Ratio (95% CI), *p*** |
| **Hct (%) at enrolment ^a^** | 0.85 (0.83, 0.88), *<0.001* |
| **Duration of fever prior to enrolment (days) ^b^** | 1.25 (1.13, 1.37), <*0.001* |
|  | **Relative change in geometric mean gametocyte density (/μl)**  **(95% CI), *p*** |
| **Hct (%) at enrolment ^a^** | -0.07 (-0.08, -0.05), <*0.001* |
| **Duration of fever prior to enrolment (days) ^b^** | 0.12 (0.07, 0.18), *<0.001* |
| Estimates derived from mixed effects linear and logistic regression adjusted for age (years) and a random effect specified for study site.  ^a^ Estimate for a 1% increase in Hct ^b^ Estimate for a one day increase in pre-enrolment fever duration | |
